# Use of Foscarnet Therapy for EBV Infection following Control of PTLD with Enhancement of Cellular Immunity in a Lung-Transplant Recipient

**DOI:** 10.1155/2011/919651

**Published:** 2011-03-03

**Authors:** Kamyar Afshar, A. Purush Rao, Vipul Patel, Kevin Forrester, Sivagini Ganesh

**Affiliations:** ^1^Division of Pulmonary and Critical Care, University of Southern California, Keck School of Medicine, 2020 Zonal Avenue, IRD 723, Los Angeles, CA 90033, USA; ^2^Department of Clinical Pharmacy and Pharmaceutical Economics and Policy, University of Southern California, 1985 Zonal Avenue, Los Angeles, CA 90033, USA

## Abstract

Posttransplant lymphoproliferative disorder (PTLD) is a serious complication following solid organ transplantation with an annual incidence rate of 3–5% in lung-transplant recipients. Pathogenesis indicates a strong association with functional over-immunosuppression and EBV infection. Clinical improvement is generally observed with reduction in immunosuppression intensity alone. We present a case of a 24-year-old woman with EBV-associated PTLD following lung transplant where decreasing the immunosuppression improved PTLD but was ineffective against controlling the EBV infection. Foscarnet in combination with immunoglobulins was successfully administered to cause a remission of the EBV infection. This is the second case reported of a persistent EBV infection after reducing immunosuppression levels and evidence of PTLD remission that required foscarnet for EBV infection control.

## 1. Introduction

Although the exact pathogenesis is uncertain, it is known that functional over-immunosuppression and Epstein-Barr virus (EBV) infections are strong risk factors for the development of PTLD. Lung-transplant recipients have at least a twofold greater risk of developing PTLD compared to other solid-organ transplants. The general therapy for PTLD includes the restoration of cellular immunity by reducing the intensity of immunosuppression. Conventional antiviral therapy with acyclovir, valganciclovir, or ganciclovir has proven ineffective, but yet remains the recommended first-line therapy for EBV infection in cases of PTLD [1]. Herein, we present a case of EBV-associated PTLD following lung transplantation showing clinical improvement of lymphadenopathy with reduction in immunosuppression intensity but having persistent EBV infection, requiring foscarnet for viral clearance.

## 2. Case Report

A 24-year-old woman underwent successful sequential bilateral living lobar lung transplantation for cystic fibrosis. EBV serology was positive for both donor and recipient. Standard triple-drug immunosuppressive medications included tacrolimus, prednisone, and mycophenolate mofetil. Four years following transplant, she experienced her first and only mild acute cellular rejection (ISHLT grade A2) that was successfully treated with a 3-day course of intravenous solumedrol (1000 mg) followed by prednisone taper. Her immunosuppressive regimen at the time included prednisone 5 mg daily, tacrolimus 2.5 mg twice daily with a therapeutic drug level of 12.4 ng/mL, and mycophenolate mofetil 250 mg twice daily. Additionally, she developed chronic kidney disease with a GFR 40 cc/min/1.73 m^2^. To preserve renal function, sirolimus was added for calcineurin-inhibitor-minimization immunosuppressive regimen. Additionally, one unit of CMV negative/leucophoresed blood was transfused for a moderate degree of normocytic/normochromic anemia (Hct 22%). The workup for blood loss had been inconclusive, and no further events occurred when seen in subsequent visits in clinic.

**Figure 1:**
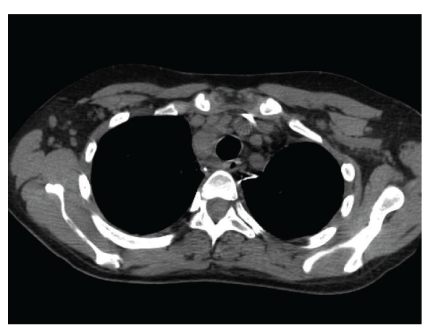
CT scan of chest demonstrated multicompartmental mediastinal lymphadenopathy, for example, a right paratracheal node measuring 14 mm in short axis.

**Figure 2:**
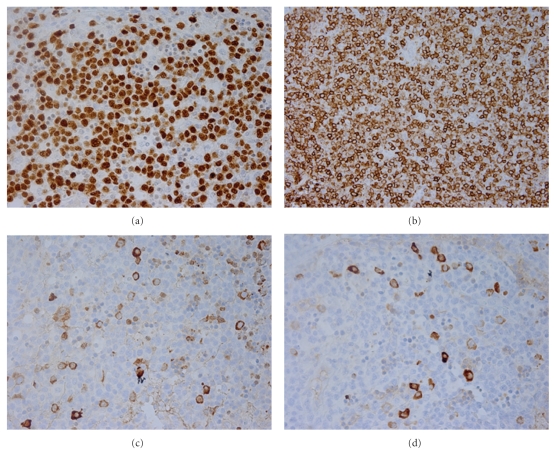
Lymph node architecture has been subtotally replaced by a diffuse proliferation of small, medium, and large lymphoid cells. (a) positive LMP-1 stain, (b) positive CD-20 antibody stain. Immunohistochemical staining with Kappa and Lambda (c and d, resp.) showed many transformed cells and was positive for plasma cells.

**Figure 3:**
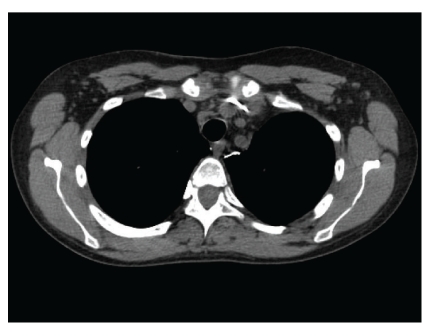
CT scan of chest posttreatment showed mediastinal nodes decreasing in size.

**Figure 4:**
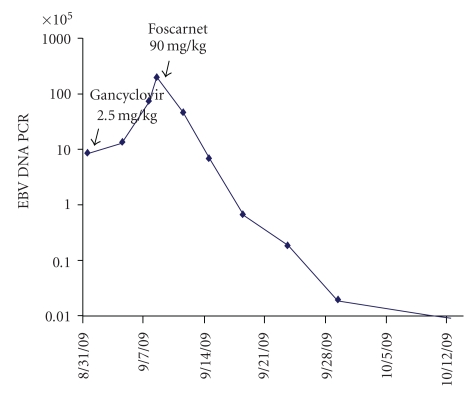
Graph indicating the EBV DNA PCR trends while on intravenous ganciclovir and foscarnet.

Six months later, she was admitted for fatigue and “B symptoms” of fevers, night sweats, and chills of three days duration. All other reviews of systems were negative. Aside from tachycardia at 110 beats/minute and febrile at 39.4 C, other vitals were normal. Physical examination was only remarkable for a palpable 2 cm  ×  2 cm right-sided firm and nonpainful cervical lymph node. Complete blood count showed pancytopenia, leucocyte count 2.4 × 10^3^ cells/mL with an absolute neutrophil count 1.6 × 10^3^ cells/mL, hematocrit 28.7%, and platelets 104 × 10^3^ cells/mL. The immunosuppression regimen included prednisone 10 mg daily, tacrolimus 0.5 mg twice daily, mycophenolate mofetil 500 mg twice daily, and rapamycin 2 mg daily. Tacrolimus and rapamycin levels were 11.4 ng/dL and 12.4 ng/dL, respectively. Empiric antibiotics were administered for potential sepsis. All final bacterial, fungal, and mycobacterial culture isolates were negative. Polymerase chain reaction (PCR) did not reveal CMV-DNA, but did demonstrate a significant number of EBV-DNA genome copies (870,908 DNA copies/mL blood). A combined approach of intravenous ganciclovir 5 mg/kg twice daily with immunoglobulin (CMV IG) administration and rapid reduction of baseline immunosuppression therapy was instituted. Both prednisone and sirolimus were tapered to 5 mg daily and 1 mg every 72 hours, respectively, giving a therapeutic drug level of sirolimus at 6.9 ng/dL. Tacrolimus and mycophenolate mofetil were completely withdrawn. CT of chest, abdomen, and pelvis revealed numerous lymph nodes in the mediastinum, cervical, and abdominal regions (Figure 1). Excisional lymph node biopsy of the right scalene lymph node was positive for polymorphic PTLD (Figure 2). The immunohistochemistry disclosed positive lymphocytes for CD-20, EBER, and EBV-LMP-1. Bone marrow biopsy was devoid of lymphoma. Intravenous ganciclovir was initiated for the control of the EBV. With the reduction in immunosuppression therapy, a desired effect of lymph node size reduction was seen on CT scan 22 days later (Figure 3). However, while on intravenous ganciclovir, PCR analysis detected continued elevation in EBV DNA levels for an additional 35 days. The peak value was 10,200,000 DNA copies/mL. Ganciclovir was changed to foscarnet 90 mg/kg. This prompted a significant reduction in EBV PCR values to undetectable levels as depicted in Figure 4. Aside from a mild increase in serum creatinine, no other adverse events occurred. During the next 9 months, all the radiographic and serologic investigations confirmed complete remission.

## 3. Discussion

PTLD is a well-recognized complication of solid organ and hematopoietic stem cell transplantation. It is a part of a group of heterogenous lymphoproliferative disorders primarily related to the oncogenic epstein-barr virus (EBV). The incidence rate of PTLD in lung transplantation is between 3–5% [2]. It generally develops within a year of transplantation but in our patient it developed 5 years after lung transplant. Overall mortality rates have been reported between 60 and 100%. Cytotoxic T-cell response is pivotal in controlling the reactivation and proliferation of the virus. EBV-infected CD20+ B cells replicate and proliferate in an environment of T-cell over-immunosuppression. Rather than undergoing lytic replication, EBV-infected cells within PTLD lesions replicate through proliferation of the transformed B cells. A constant degree of excessive immunosuppression allows for proliferation of PTLD B-cell lymphocytes to manifest in an aggressive monoclonal or polyclonal B-cell lymphoma [3]. 

Besides impaired T-cell immunity, other risk factors of developing EBV infection following lung transplant include donor or recipient EBV seropositivity, a natural primary transmission, and HLA-A3 expression. The highest risk groups are patients with EBV seroreactive mismatch (D+/R−). This group has a 30-fold higher risk compared to the seronegative group (D−/R−). Molecular analysis, however, shows that EBV-associated PTLD following lung transplant more commonly originates from the recipient [4]. There is currently debate over acquiring EBV infection through transfusion-related sources. Some reports have shown that EBV can be transmitted and induce infectious mononucleosis if the donors were viremic for EBV at the time of donation [5]. Symptoms would develop 5–7 weeks after blood product transfusion. However in a study by Wagner et al., the effects of transfusing 15 EBV-seropositive blood products in 15 EBV-seronegative patients were assessed, and the results did not indicate EBV transmission [6]. Nevertheless, it is prudent to avoid the risks of transfusing blood products that are known to be contaminated with EBV. 

Due to its rare occurrence, it is challenging to make definitive therapeutic recommendations for PTLD. Treatment strategies are primarily based on anecdotal reports. Comparative data to evaluate treatment options are currently lacking. The cornerstone of therapy is the reduction or complete withdrawal of immunosuppressive therapy for enhancement of cellular immunity. This allows the normalization of the T-cell immunity and reduces proliferation of the transformed B cells. Of course, this is in concert with balancing the risk of allograft rejection. In the management of EBV-associated PTLD, the control of the EBV infection may or may not play a pivotal role, but antiviral therapy has been advocated by some centers [7]. European best practice guidelines recommend the use of antivirals for at least 1 month after the diagnosis of PTLD [8]. Acyclovir, valganciclovir, or ganciclovir are first-line therapeutic recommendations. When monitoring the EBV PCR values in association with PTLD, the DNA levels generally fall within 2 weeks and become undetectable by reducing immunosuppression and initiating antiviral therapy [7, 9]. Patients have been reported to have peak plasma EBV DNA PCR levels of 663,000 copies/mL [9]. In our case, it was clearly evident that clinical response of PTLD was effectively attained with reduction in the immunosuppression regimen, but DNA copies of EBV by PCR were persistently detected at high levels weeks after. 

Controlling EBV infection is generally successful with nucleoside analogues, but EBV viral loads can be exceedingly high despite the continuous administration of intravenous ganciclovir in patients with PTLD. The mechanism of action of nucleoside analogues, such as ganciclovir is the inhibition of *viral* DNA polymerase and affecting cells that undergo lytic cycle. Their use in EBV-infected PTLD cases is of limited value because as many as 90% of EBV-infected PTLD lesions are transformed cells that do not undergo lytic replication. Foscarnet is distinct from the nucleoside analogues such that it does not require viral enzyme activation. Therefore, foscarnet was a logical alternative based on clinical assessment. Its use has been reported in another patient with EBV-associated PTLD who received a heart and kidney transplant (10). That patient had a successful and persistent 10-month remission of the disease with reduction in the level of immunosuppression and a 4-week foscarnet therapy. Similar to our case, anti-CD 20 monoclonal antibodies or chemotherapeutic agents were not required for the resolution of the PTLD and EBV infection.

## 4. Conclusion

EBV-associated PTLD typically occurs in the setting of profound immunosuppression. The resulting impairment of EBV-specific cytotoxic T lymphocytes (CTLs), donor and recipient seropositivity, and HLA-A3 expression and primary transmission are the primary risk factors for developing PTLD. It is challenging to standardize a comprehensive treatment strategy due to PTLD rare occurrence. A rapid reduction or complete withdrawal of immunosuppression is the hallmark of therapy. Antiviral therapy to control EBV infections is controversial, but still advocated. Nucleoside analogues such as acyclovir, valganciclovir, or ganciclovir are of limited value in EBV-associated PTLD due to the proliferation of the cells remaining in latent form and requiring *viral* enzyme activation. 

Generally, EBV PCR values are undetectable with clinical remission of PTLD. Our patient showed significant improvement of PTLD features by merely decreasing immunosuppression levels. However, the EBV PCR values continued to escalate while receiving nucleoside analogue against persistent EBV infection following clinical remission of lymphadenopathy. The use of foscarnet in combination with immunoglobulins resulted in successful reduction in EBV PCR values. This is only the second case reported of a persistent and successful remission of EBV infection and PTLD. Further analysis is warranted to assess foscarnet's role as a first-line agent for the control of EBV associated with PTLD.
